# Family–School Partnership as Interpretive Practice: Educator Accounts of Attribution, Communication, and Decision-Making in Arab Education in Israel

**DOI:** 10.3390/bs16101871

**Published:** 2026-10-10

**Authors:** Sawsan Awwad-Tabry

**Affiliations:** Faculty of Graduate Studies, Oranim Academic College of Education, Kiryat Tiv’on 36006, Israel; sawsan_t@oranim.ac.il

**Keywords:** family–school partnership, educator accounts, interpretive practice, inclusive education, Arab education in Israel, reflexive thematic analysis, qualitative research

## Abstract

Family–school partnership is often discussed in relation to student support, yet adult interviews primarily illuminate how educators interpret family knowledge and organize professional action. This interpretivist qualitative study examined how 13 educators in Arab education in Israel constructed partnership when interpreting students’ learning, emotional, social, or behavioral difficulties. The educator interviews were analyzed using topic-focused reflexive thematic analysis. Four unmatched mother interviews, collected to explore school communication, transparency, and decision inclusion, received a separate, bounded contextual reading; they neither generated themes nor triangulated educator accounts. Three educator-derived themes concerned changing the terms of engagement before crisis, treating family knowledge as capable of revising professional attributions and informing response, and attributing participation- and belonging-related meanings to coordinated adult support. A descriptive cross-case display showed how reattribution-and-response accounts were distributed across the educator interviews, including partial, ambiguous, and negative cases; these counts describe this dataset rather than estimate prevalence. The mother accounts complicated school-centered constructions by describing contact that could remain delayed, one-directional, or without influence over decisions. The findings support an adult-centered account of contact, epistemic authority, communicative accessibility, and decision inclusion; they do not demonstrate a causal pathway or student outcomes.

## 1. Introduction

### 1.1. Educator Interpretation in Contexts of Student Difficulty

Educators interpret learning, emotional, social, or behavioral difficulties within settings where students may also encounter exclusion, stigma, constrained participation, and weakened school belonging. Research involving students with special educational needs and social, emotional, or behavioral difficulties emphasizes the relational and institutional character of these experiences (Carmona & Montanero, 2025; Enderle, 2025), while special educators themselves describe tensions between support responsibilities and school structures (Bettini et al., 2026). This scholarship provides the context for the present inquiry; the empirical focus is educators’ meaning-making and professional action.

In this study, “students receiving inclusion support” refers broadly to students in mainstream or integrated settings who receive additional educational, emotional, remedial, or special-education support. Belonging was used only as a sensitizing concept: prior scholarship describes it in terms of acceptance, respect, inclusion, and connection and links it to engagement and psychosocial adjustment (Baumeister & Leary, 1995; Goodenow, 1993; Arslan, 2021; Štremfel et al., 2024; Wilkins et al., 2023). The analysis therefore examines how educators invoked belonging and related constructs when explaining their practice.

### 1.2. Family–School Partnership and Inclusive Education

Family–school partnership scholarship emphasizes communication, involvement, decision-making, and shared responsibility (Epstein, 1995, 2018). In inclusive and special-education settings, partnership is commonly distinguished from occasional school contact by reciprocal dialogue, recognition of family knowledge, and coordinated support. Research has examined welcoming school cultures, parent–teacher collaboration, communication, and family–school interventions (Bachman et al., 2024; Dan et al., 2023; Grüter et al., 2025; Leenders et al., 2019; Smith et al., 2020). The present study builds on this literature by examining how educators constructed the adult interpretive and decision-making work of partnership.

Critical and relational scholarship complicates partnership as a neutral exchange of information. Deficit thinking can locate difficulty in a student, family, or community while leaving institutional practice unexamined (Valencia, 2010). Family–professional collaboration can also reproduce unequal cultural and epistemic authority (Kalyanpur & Harry, 2012), and parent–teacher conferences can negotiate whose knowledge carries the right to define a problem or shape a decision (Caronia, 2024). Culturally responsive engagement instead positions families as cultural brokers and co-constructors of meaning (Ishimaru et al., 2016).

Terminology is used consistently throughout the manuscript. Contact refers to the occurrence and timing of an interaction between school and family, regardless of reciprocity. Communication refers to the transmission or exchange of information through a particular channel. Family–school partnership denotes a relational process in which family knowledge can enter professional interpretation or influence action. Co-interpretation is the narrower process through which family knowledge revises, extends, or confirms an educator’s formulation of difficulty. Decision inclusion refers to an opportunity for family input while interpretations and decisions remain open, rather than notification after closure. These distinctions organize the analysis of adult attribution and professional response.

### 1.3. Arab Education in Israel as a Minority Education Context

Arab education in Israel forms part of the national education system while operating largely as a distinct Arabic-medium minority sector. Scholarship describes Arab citizens’ position as a national minority, persistent institutional inequalities, and culturally and institutionally situated family–school relations (Arar et al., 2014). Research in Arab and Israeli education has also examined parental inclusion in decisions, parent–teacher helping relations, institutional trust, and barriers to inclusive education (Grinshtain & Harpaz, 2023; Jorban et al., 2024; Zedan, 2012). These sources establish the wider setting; they are not evidence that every feature they describe was experienced by participants in this study.

Closely related work by Jorban et al. (2024) interviewed 20 parents of children with special educational needs in the Arab community of Israel and found that parents experienced exclusion from school decisions, including being informed of outcomes rather than consulted. The present study differs in question, evidence, and analytic logic: it examines educators’ interpretive work through reflexive thematic analysis. The four mother interviews reported here are too small and unmatched to corroborate either Jorban et al. or the educator themes. Their descriptions of being a “spectator from the side” or separated by a “glass wall” are instead read alongside that literature as a bounded contextual reminder that contact may transmit information without creating decision inclusion.

Structurally, Arab education is not an independent private system. It includes official and recognized but non-official schools supervised by the Ministry of Education, with Arabic as the principal language of instruction (Central Bureau of Statistics, 2018). Schools teach core secular subjects shared across the national system alongside sector-specific Arabic-language and Arab/Palestinian history and identity content; Hebrew and Jewish history are also taught (OECD, 2026). Teaching staffs are predominantly Arab, although cross-sector employment is legally possible and occurs to a limited extent (Maagan & Blass, 2026).

The study did not compare Arab and Hebrew education, ask systematically about minority positioning, or empirically test the effects of shared language, community proximity, governance, or staffing. Those features are therefore treated as contextual conditions drawn from scholarship and system description, not as findings. The empirical analysis makes narrower contextual claims only where participant material supports them: concerns about diagnosis and labeling, accessibility of professional communication, mistrust, and inclusion in school decisions.

### 1.4. The Present Study

Research on family–school partnership has established the importance of communication and involvement, but a more specific interpretive question remains: how do educators describe family knowledge entering, revising, or being excluded from professional interpretation and decision-making? This adult-centered question concerns attribution, communication, epistemic authority, and the organization of support.

Accordingly, this study asks: How do educators in Arab education in Israel construct the meaning of family–school partnership when interpreting students’ learning, emotional, social, or behavioral difficulties and organizing support? The primary analysis draws on 13 educator interviews. Four unmatched mother interviews are retained separately as bounded contextual accounts of communication and decision inclusion.

## 2. Materials and Methods

### 2.1. Research Design

This study used an interpretivist qualitative design and topic-focused reflexive thematic analysis (RTA) to analyze 13 educator interviews (Braun & Clarke, 2006, 2019, 2021). The analytic object was not an objective mechanism linking partnership to student outcomes, but the patterned meanings through which educators described partnership, interpreted difficulty, and justified or revised professional action. Interviews were understood as situated accounts produced through the interaction of the guide, participants’ experiences, and the researcher’s interpretive engagement.

The design was topic-focused and conceptually sensitized from the outset by family–school partnership, inclusion, emotional support, and belonging. It was therefore neither wholly inductive nor a theory-testing exercise. Reflexive thematic analysis was used to move beyond listing the interview topics toward an interpretation of how adult positioning, attribution, and response were connected in participants’ accounts.

### 2.2. Participants and Sampling

The primary dataset comprised 13 educators from Arab education contexts in Israel (Table 1). Four mothers were interviewed separately and used only as a small contextual counter-perspective. They were not a representative parent sample, were not matched to the educators or students discussed in the educator interviews, and were not used to validate, corroborate, or increase the credibility of educator claims.

The educator group included inclusion, special-education, remedial-instruction, homeroom, English, and general-education teachers working across elementary-, middle-, and high-school settings. This heterogeneity was purposive: the analysis sought patterned meanings across educators with direct experience of inclusion support, not role-specific or school-level comparisons. It consequently supports no claim that a theme characterizes a particular professional subgroup. Three mothers had daughters in technological-school settings; the fourth daughter’s school type was not documented.

Sample adequacy was considered across the five dimensions of information power proposed by Malterud et al. (2016). First, the study aim was narrow: it concerned how educators constructed partnership when interpreting difficulty and organizing support. Second, sample specificity was moderate rather than high. All participants met a focused experiential criterion, but they were heterogeneous in role and school level. Third, theoretical support was also moderate: partnership, inclusion, and belonging scholarship sensitized the inquiry without functioning as a deductive model. Fourth, dialogue quality was assessed from the detailed, case-based material generated through follow-up in 60–90-min interviews, rather than inferred from duration alone. Fifth, the analysis sought a cross-case interpretive account of shared meanings, not prevalence estimates, subgroup comparison, or case-level theory building. Taken together, these dimensions were judged to provide adequate information power for developing three bounded themes from 13 educator interviews, while the sample’s heterogeneity limits claims about particular professional groups.

Consistent with RTA, saturation was not used as a stopping rule or proof of completeness (Braun & Clarke, 2021). The information-power appraisal supports development of a bounded interpretation from these 13 educator interviews. Descriptive counts later reported for one analytic pattern map the distribution of account forms within this dataset; they do not estimate prevalence, compare subgroups, or determine theme importance.

### 2.3. Recruitment and Field Access

Recruitment combined purposive and snowball strategies. Study invitations were posted in professional groups for educators in Arab education in Israel on social media platforms (Facebook and Instagram), rather than sent through the researcher’s personal contacts, and some recipients forwarded them to other eligible adults. The researcher had no prior professional or personal relationship with participants, participating-school principals, or recruitment intermediaries; no participant was the researcher’s student, employee, or supervisee. Invitations described the study purpose, voluntariness, confidentiality, and withdrawal rights and stated explicitly that participation or refusal would have no professional consequences. This separation was important because recruitment through personal networks can otherwise create perceived obligations or favor socially desirable participation.

Participants were affiliated with six Arab school settings spanning elementary-, middle-, and high-school (including technological) contexts; schools were not analyzed as institutional cases. Ethical approval was obtained from the Institutional Review Board of Oranim Academic College of Education, and access to school-based participants was coordinated with principals where relevant. Because participants were recruited individually as adults, no separate Israeli Ministry of Education authorization was sought. To reduce socially desirable portrayals of partnership, interviews asked not only about desired practices but also about challenges, unsuccessful contact, disagreement, and non-disclosure. Analysis treated benefit-oriented accounts as situated responses to the guide and retained ambiguous, partial, and negative cases. These procedures cannot eliminate desirability bias, but they make its likely direction and analytic treatment explicit. School, student, and locality identifiers are not reported.

### 2.4. Data Collection

Data were collected through semi-structured, in-depth interviews conducted between February and April 2025. Interviews lasted approximately 60–90 min and were conducted in Arabic, the participants’ native language. Interviews took place in settings suited to participants and the research context, including schools, participants’ homes, and Zoom.

A semi-structured interview guide provided a common topical frame while allowing follow-up of participants’ examples and meanings (Brinkmann & Kvale, 2015; Creswell & Poth, 2018). Educator interviews addressed communication, trust, parental involvement, shared responsibility, decision-making, and challenges in supporting students with learning, emotional, social, or behavioral difficulties. Four mother interviews were collected within the broader qualitative inquiry to obtain parent-side accounts of school communication, transparency, involvement in decisions, and conditions that might help parents feel more involved. They were not recruited as matched counterparts to educators and were not intended as a representative or coequal parent sample.

Representative educator questions included: “How do you describe your relationship with the parents of students receiving inclusion support?”; “What expectations do you have of parents regarding educational partnership?”; “How does partnership with parents contribute to students’ sense of belonging, emotional support, or participation in school?”; and “What challenges make family–school partnership difficult?” The third question presupposed a potentially positive family–school partnership and likely increased the salience of benefit-oriented accounts. The study is therefore not presented as inductively discovering that partnership contributes to belonging. Instead, the analysis examines how participants constructed, qualified, and sometimes destabilized that proposed relationship. Representative mother questions concerned relationships with school, involvement in decisions, transparency, and what would help them feel more involved.

The interviews were audio-recorded with participants’ consent and transcribed verbatim. Identifying details were removed, and transcripts used a uniform coding system in which M referred to teacher/educator interviews and P to parent/mother interviews. Educator coding, memoing, and theme development, and the focused contextual review of mother transcripts, were conducted in Arabic; only quotations selected for reporting were translated into English. This preserved interpretive nuance and reduced loss across languages (van Nes et al., 2010). Translation attended to intended meaning, emotional tone, and culturally situated expressions, and each reported translation was checked against the corresponding Arabic recording or transcript. Where an educator briefly voiced a student’s felt sense in the first person within an otherwise third-person account, single quotation marks preserve the educator’s wording rather than imply direct student testimony (e.g., M02 in Section 3.3).

### 2.5. Data Analysis

The 13 educator interviews were analyzed manually using topic-focused RTA (Braun & Clarke, 2006, 2019, 2021). Recursive reading, coding, memoing, and theme development attended first to descriptions of contact, family knowledge, diagnosis, non-disclosure, support decisions, and student behavior. Subsequent analysis asked whom accounts positioned as knowledgeable, how difficulty was attributed, what professional action was described, and where communication failed to become partnership.

After the three educator-derived themes had been developed, all four mother transcripts were reread separately through a bounded, question-focused contextual analysis of reciprocity, interpretive voice, and decision inclusion. They were not blended into the educator code frame or cross-case display, did not generate a second set of themes, and did not count as confirmation or triangulation. All four mothers are represented in Section 3.4: P01 and P03 are quoted because their “spectator” and “glass wall” formulations most directly and self-containedly express contact without influence, while P02 and P04 are summarized. Selection reflected relevance and clarity for this bounded question, not frequency, representativeness, or convergence with educator claims.

Several interpretive decisions moved the analysis beyond the guide’s topics. Codes concerning WhatsApp updates, positive contact, pre-crisis calls, and flexible channels were interpreted as changing the terms of engagement because timing, purpose, and educator stance distinguished a summons after difficulty from an invitation into an ongoing relationship. Parent knowledge was likewise examined as a possible change in professional interpretation, not merely as information exchange.

After Theme 2 had been constructed interpretively, a cross-case descriptive display was developed to test its boundaries and show how the proposed account was distributed across all 13 educator interviews. Two criteria were applied: whether an educator narrated family information followed by a changed or confirmed understanding of difficulty, and whether the same account then described a concrete changed response. Both criteria were derived from the analytic definition of Theme 2 after the theme had been developed, rather than specified in advance. The strict criterion required a revised understanding; the broader criterion also admitted M01’s confirmatory case.

Ambiguous, partial, and negative cases were retained rather than absorbed into the pattern: M05 described a changed response without a specified reattribution; M11 described reattribution without a specified response; M04, M08, M09, M12, and M13 supplied no qualifying family-information episode, with M12 excluded because the information came from the student. The resulting counts describe the distribution of account forms in this bounded dataset. They are not prevalence estimates, a threshold for theme importance, or a reliability result. The display is conceptually consistent with RTA because it does not treat coding as objective measurement or replace interpretive theme development; it makes the claim’s scope and limits visible.

An early outcome-oriented grouping linked coordination with confidence, security, participation, and belonging. Reflexive review showed that the interviews could not sustain those psychological states as findings. The final third theme therefore treats the connection itself as an educator attribution and separates described actions or observations from meanings educators inferred for students.

A further interpretive choice concerned “parental resistance.” Educator accounts were read alongside M03’s initially inaccessible written communication, M08’s concern that parents may be experienced as judges, and mothers’ descriptions of after-the-fact decisions. This supported a relational reading in which friction could involve family fear or non-disclosure, educator defensiveness, inaccessible communication, or school-defined decision-making. Memos recorded code clusters, candidate theme labels, alternative readings, and evidentiary boundaries. Educator quotations were selected to show each interpretive claim and its limits; mother-quotation selection followed the separate procedure described above. Themes remain researcher-developed constructions, not discoveries independent of the research question and analytic position.

### 2.6. Researcher Positionality and Reflexivity

The sole researcher is a Palestinian Israeli Arab education scholar with professional and scholarly familiarity with Arab education in Israel, inclusion, and culturally situated educational practice. This proximity supported contextual and linguistic understanding but also made familiar explanations—particularly the presumed value of partnership and the cultural salience of stigma—easy to take for granted.

The researcher had no prior relationship with participants or recruitment intermediaries and no educational or employment authority over participants. Even so, shared cultural and professional context, open circulation of invitations on social media, and the guide’s positive interest in partnership could facilitate rapport while also encouraging socially desirable accounts. Reflexive analysis therefore treated benefit-oriented narratives as invited, situated constructions and deliberately examined challenge-focused, partial, and negative cases. M03, M08, and the mother interviews disrupted a simple “communication is beneficial” reading and supported the distinctions among contact, communication, co-interpretation, and decision inclusion.

Because analysis was conducted by one researcher, there is no claim of coder consensus or interpretive neutrality. Reflexive memos tracked the guide’s assumptions, the researcher’s interpretations, plausible alternatives, and decisions about evidentiary boundaries. The absence of prior relationships, the explicit assurance that participation or refusal had no professional consequences, confidentiality protections, and challenge-focused questions reduced some pressures on disclosure but cannot remove self-presentation or social desirability.

### 2.7. Analytic Quality and Transparency

Analytic quality was pursued through conceptual coherence among the interpretivist design, reflexive thematic analysis, research question, claims, and evidence (Braun & Clarke, 2021). The analysis distinguishes descriptive topics from interpretive themes, shows how alternative readings were considered, identifies the unequal status of the two datasets, and specifies where participant accounts support only adult-level interpretations.

Transparency was supported by preserving source files, participant codes, coding documents, analytic tables, and memos linking candidate themes to extracts. These materials organized the researcher’s interpretive process; they are not presented as an objective audit trail that could reproduce a single correct analysis. Member checking and inter-coder agreement were not used because the themes were treated as situated researcher constructions rather than participant-validated summaries.

The quality claim is consequently bounded: the analysis offers a coherent and evidenced interpretation of these interviews. It does not claim exhaustive coverage, saturation, cultural representativeness, corroboration across independent datasets, or validation of student outcomes.

### 2.8. Ethical Considerations

The study was approved by the Institutional Review Board of Oranim Academic College of Education (protocol code 106, approval date 10 June 2024). All participants provided informed consent before participating in the interviews. Participation was voluntary, and participants were informed of their right to decline to answer any question or withdraw from the study at any stage without consequence. To protect confidentiality, identifying details were removed or masked in transcripts and reporting, and participants are referred to by code only. Audio recordings and transcripts were stored securely and were accessible only to the researcher.

## 3. Results

Analysis of the 13 educator interviews generated three themes concerning adult practice: changing the terms of engagement; family knowledge, reattribution, and response; and educators’ attribution of meaning to coordinated support. Students were neither interviewed nor observed. Accordingly, descriptions of student confidence, security, participation, or belonging are reported only as meanings educators assigned retrospectively, not as findings about students’ psychological experience or effects of partnership.

The mother interviews did not contribute themes or equivalent evidence. Section 3.4 reports their bounded contextual reading of reciprocity and decision inclusion after the educator results.

The three educator-derived themes are: (1) From Crisis Contact to Relational Trust: Changing the Terms of Engagement; (2) Parents as Co-Interpreters: Family Knowledge, Reattribution, and Response; and (3) Coordination as an Educator Attribution: Adult Alignment, Visibility, and Participation (Table 2).

### 3.1. From Crisis Contact to Relational Trust: Changing the Terms of Engagement

Educators distinguished contact that occurred after a problem from contact intended to alter the relationship before crisis. What changed was not simply communication frequency. Participants described changing the timing, purpose, content, channel, and relational stance of contact: initiating it early, including positive information, asking for family knowledge, and adapting the medium to parents’ access.

One teacher described this deliberate repositioning:

“I do not wait for something to happen in order to speak with the parent, but create proactive communication already in the first weeks. Sometimes it is a phone call, sometimes a WhatsApp message, sometimes a small request: ‘Could you send something small that the child likes, so that I can integrate it in the classroom?’ This is a way to make the parent feel that he is being listened to, that he is present, even from afar.”

(M10, teacher)

The request for something the child liked changed the parent’s position from recipient of a school report to contributor of knowledge. The evidence supports an educator action—inviting information before difficulty—not the parent’s actual feeling of being heard.

M08 made the educator’s own positioning especially visible:

“A successful educational partnership is not built from reminder messages or contact only when a problem has arisen—it is built from a school culture that believes in parents as partners and not as judges.”

(M08, inclusion teacher)

The phrase “not as judges” reveals potential educator defensiveness as part of the interaction. In this account, a crisis-oriented or evaluative stance can make contact protective of the school rather than dialogic. Educators described countering that stance through positive updates, regular calls, and channel flexibility. M07 referred to weekly messages or monthly meetings, while M05 described tailoring contact to telephone, text, WhatsApp, or email.

The theme therefore concerns a shift in educators’ terms of engagement: from summoning a parent to explain a problem toward inviting a parent into an interpretive relationship. The interviews do not show whether parents consistently experienced these initiatives as trusting or non-pressuring; that remains outside the evidence.

### 3.2. Parents as Co-Interpreters: Family Knowledge, Reattribution, and Response

In retrospective accounts, educators sometimes narrated family information, a changed or confirmed understanding, and a changed response in that order. The cross-case descriptive display (Table 3) applies the criteria specified in Section 2.5 to all 13 educator interviews. Five accounts described both elements under the strict criterion (M02, M03, M06, M07, M10), and a sixth did so under the broader criterion that admits M01’s confirmatory understanding. M05 described a response without specified reattribution, M11 reattribution without a specified response, and five interviews no qualifying episode.

M03 provides the most behaviorally specified retrospective account. The teacher first described a mother who appeared “disconnected” from her child’s learning and then reported learning that she could not read or write. The teacher next described replacing written messages with supportive telephone conversations and later reported greater maternal involvement. The interview preserves this narrated order: family information, reattribution, changed channel, and later adult participation.

M10 narrated the pattern vividly around a student initially framed in deficit terms. A student who threw objects, refused lessons, and had moved through several schools was, in M10’s words, regarded as a “lost case.” M10 asked the guardian directly:

“What do you see in this child at home that I still do not see?”

(M10, teacher)

The guardian described the student caring for a younger sibling, cooking, and writing poetry. M10 then summarized the stance, “You know him better than I do; help me see,” and subsequently described building a different relationship and assigning a classroom responsibility. Within the educator’s account, the family information broadened which capacities counted as relevant.

Two further interviews narrated comparable ordering around emotional and behavioral difficulty. M06 reported:

“During conversations with the parents, I discovered that there were pressures at home that made it difficult for him to concentrate. Together we formulated an action plan that included consistent work both at home and at school, while setting small and positive goals.”

(M06, teacher)

M07 likewise described learning of “a complex emotional situation at home” and then reported that the adults developed “a unified response system for frustrating situations,” including a shared calming cue and a quiet space. In M06 and M07, family information was narrated before a re-situated understanding and a changed plan.

M02’s account, reported more fully in Section 3.3, also meets the descriptive criteria: the educator narrated parents’ disclosure of a strength in technology and programming, a broader picture of the student, and a corresponding classroom opportunity in that order.

M01’s interview illustrates a related but distinct pattern: confirmatory rather than revisory reattribution. A Grade 10 student showed avoidance, a marked lack of confidence, and somatic signs before tests. M01 reported:

“After several weeks I contacted her parents, and in my meeting I was surprised to discover that at home, too, they saw similar symptoms, but the parents thought it was simply shyness.”

(M01, teacher)

The exchange did not overturn M01’s prior interpretation so much as confirm it against family observation and jointly reframe it as anxiety. M01 then described a specific change in classroom practice:

“From my side, I created a safe space for her in the classroom. I let her choose when and how she participated, did not pressure her to speak in the group, and allowed her to express herself in writing when speaking was difficult.”

(M01, teacher)

The interview also reported later participation: an initial hand-raise, then reading aloud, then speaking in a small group. Because M01 had already suspected anxiety before the family conversation, this case is included under the broader, confirmatory criterion rather than the strict criterion used for the preceding five cases.

Two further interviews described only one element of the proposed account pattern and mark its boundary. M05 expressed a general positioning of parents as contributors to strategy formation:

“I see parents as co-educators. They know their child best, and their insights are invaluable in creating realistic and effective strategies.”

(M05, special education teacher)

In a specific case, M05 narrated a mother’s disclosure of what worked at home and then a changed learning plan for a student with dysgraphia. The account specifies a changed response but not a corresponding change in how M05 understood the difficulty itself; family knowledge informed practice without a described reattribution. M11 shows the inverse pattern. Family information introduced sensory sensitivity, routines, and a connection to music into an interpretation previously organized around classroom difficulty:

“She told me about small rituals they have, about his sensitivity to noise, about his connection to music. It changed the way I approached him. I understood at that moment that a parent is not an ‘accompanying factor’; he is a source of emotional knowledge that is not found in any report.”

(M11, teacher)

The account supports a change in educator attribution, but the interview does not specify what the changed approach consisted of in practice, so M11 is not counted among the fully specified cases.

The remaining five interviews (M04, M08, M09, M12, M13) discussed family knowledge and communication in general terms, valuing parents as informants about a student’s history, personality, or home environment, without linking a specific disclosure to a specific change in interpretation or response. A superficially similar case in M12 described a comparable change in support following a disclosure of difficulties at home, but the disclosure came from the student directly rather than a parent or family member, and so falls outside this theme’s definition of family knowledge. M01’s interview also illustrates a further limit of co-interpretation, separately from the case above: M01 reported that non-disclosure of diagnoses, emotional problems, or medical information constrained staff planning in another case. The interview supports the educator’s experience of missing information; it cannot establish why a particular parent withheld it. Treating non-disclosure automatically as denial would reproduce the deficit reading that the theme otherwise questions.

Parents as co-interpreters therefore names an educator-constructed shift in whose knowledge could revise or confirm a professional explanation and inform action. Both elements were described in five accounts under the strict criterion and six under the broader criterion; two additional accounts were partial, and five supplied no qualifying episode. This distribution bounds the interpretive claim.

### 3.3. Coordination as an Educator Attribution: Adult Alignment, Visibility, and Participation

Educators retrospectively connected coordinated adult action with changes they observed or inferred around students. This theme analyzes that attribution rather than treating coordination as a mechanism operating on students. It separates described adult actions and observable participation from psychological meanings assigned by educators.

M06 expressed the proposed relationship directly:

“When the student sees that the teacher and the parent speak with each other, exchange information, and coordinate goals, he understands that he is important. This feeling, that he is at the center, strengthens his self-confidence and his feeling that he is part of the class, even if he has difficulties.”

(M06, teacher)

The quotation provides evidence that this teacher interpreted adult coordination as a signal of importance.

M02 described more observable adult and student actions. After learning from parents about a student’s strength in technology and programming, the teacher created an opportunity for the student to contribute to a school project; the parents encouraged the student at home; and the teacher observed the student present the work in class:

“This feeling, that ‘people believe in me at home and at school,’ gave him tremendous confidence. When he presented his work in class, his face lit up. Suddenly he felt he belonged, was important, part of the group.”

(M02, teacher)

The presentation is an educator-reported behavior, while confidence and belonging are the teacher’s interpretations. Analytically, the account places family information, a changed opportunity, parental encouragement, and observed participation in that order.

Across the educator dataset, coordination was narrated through similar distinctions between adult action and inferred student experience. Participants described exchanging information, aligning expectations, recognizing strengths, or creating opportunities to participate; they then interpreted these actions as conveying visibility, consistency, or belief. The study’s finding is this educator attributional model.

### 3.4. Contextual Counter-Perspective from Four Mothers: Contact Without Influence

Following development of the educator themes, all four mother accounts were read for the bounded question of whether school contact offered reciprocal communication, interpretive voice, and decision inclusion. This subsection is not a fourth theme or a second thematic dataset. P01 and P03 are quoted because their concise “spectator” and “glass wall” formulations most directly express contact without influence; P02 and P04 are summarized so that all four accounts remain visible. Because the mothers were few and unmatched, their accounts contextualize a possible counter-position but neither triangulate nor validate the educator themes.

Educators described parental resistance to diagnosis, non-disclosure, limited availability, and unsuccessful contact. Earlier analysis risked resolving these difficulties too quickly through fear, stigma, or care for the child. Those motives are educator interpretations. The material also locates friction in school practice: M03 initially used an inaccessible written channel; M08’s “parents as judges” formulation disclosed educator defensiveness; and M05 reported reframing diagnostic recommendations as “support strategies for success,” indicating that professional language itself shaped the encounter.

The mother accounts added a limited view of contact without influence. P01 described being informed after events or decisions:

“There are quite a few moments when I feel like a ‘spectator from the side’—that I am not really part of the process, but rather receive updates when something has already happened.”

(P01, mother)

This account does not refute a particular educator because the datasets are unmatched. It does, however, complicate the assumption that school-initiated communication constitutes partnership: information may be transmitted while the parent remains outside interpretation and decision-making.

P03 similarly described “a glass wall” between parents and staff. P02 portrayed herself as simultaneously carrying parental, counseling, and homeroom functions, and P04 wanted parents treated as part of the educational fabric. Read modestly, these accounts identify a counter-position in which contact can be visible and frequent but still experienced as technical, delayed, or without influence.

The combined material supports friction rather than a uniform success narrative. Breakdown was relationally distributed across family non-disclosure or disagreement as narrated by educators, educator assumptions, inaccessible channels, diagnostic framing, and school-defined decisions. The data do not show whether proactive communication sometimes became pressure, whether educators disagreed systematically about appropriate parental involvement, or whether parental resistance was specifically grounded in distrust of a school’s interpretation. These remain questions generated by the analysis, not findings supplied by it.

In summary, educator and mother materials remain analytically distinct: the former generated themes about professional interpretation, while the latter show why contact cannot be assumed to confer epistemic or decision-making influence.

## 4. Discussion

### 4.1. Partnership as Epistemic and Interpretive Practice

The contribution is located at the adult level. Educators described the terms of contact, the entry of family knowledge into professional interpretation, changed or confirmed attributions, and responses they later reported; student-related meanings remained retrospective educator interpretations. The more distinctive issue is epistemic authority: whether family knowledge is treated merely as information received, as knowledge capable of revising a professional formulation, or as input excluded once an interpretation or decision has closed.

This reading refines rather than replaces Epstein’s (1995, 2018) dimensions of communication, involvement, decision-making, and shared responsibility. The cross-case display specifies how often different account forms appeared in this dataset without converting theme construction into prevalence measurement. Family information was not inherently partnership; its analytic significance depended on whether educators described it entering their reasoning or informing action.

The distinction among contact, communication, and partnership is therefore central. Contact can occur without reciprocity; communication can transmit information without redistributing interpretive authority; partnership requires some permeability of professional understanding or decision-making to family knowledge. The mother accounts make the difference visible by describing contact that occurred after decisions or did not confer influence.

This distinction connects the accounts to scholarship on unequal epistemic authority and deficit thinking (Caronia, 2024; Kalyanpur & Harry, 2012; Valencia, 2010). The study identifies a recurring narrated configuration in which educators placed family information, changed or confirmed understanding, and a reported response in that order, while partial and negative cases show that the elements need not co-occur.

### 4.2. When Contact Becomes Partnership: Institutional Conditions

Across the accounts, contact approached partnership under three institutional conditions: it occurred while an interpretation or decision remained open; the communication channel was accessible and reciprocal; and family knowledge was permitted to alter a formulation or action. Contact remained information transmission when it was late, technical, inaccessible, or delivered after decisions had closed.

These conditions direct attention to institutional control. Schools commonly control the timing and agenda of contact, the terminology and channel through which a concern is presented, and the point at which a decision is treated as settled. M03’s change of channel, M08’s image of parents as judges, and the mothers’ “spectator” and “glass wall” formulations show different ways that these arrangements can enable or constrain influence. Family willingness alone is therefore an insufficient account of partnership. Comparable institutional limits appear in research with foster parents in Israel, whose relationships with school staff varied in quality and who described feeling unseen by the system (Awwad-Tabry, 2024).

Family influence over decisions is not equivalent to the existence or frequency of contact. Decision inclusion was present, as an analytic possibility, when family input could enter while educators were still interpreting a difficulty or selecting a response; it was absent when families were notified only after closure. The mother accounts illuminate this distinction contextually without serving as corroboration of any educator episode.

The analysis also identifies unanswered boundary conditions. Participants did not directly describe proactive contact as intrusive, systematically debate appropriate limits of parental involvement, or provide paired cases of distrust in a school’s interpretation. The three conditions are therefore an interpretive synthesis of these accounts, not an implementation model or exhaustive definition of ideal partnership.

### 4.3. Friction and the Uneven Enactment of Partnership

No participant rejected partnership as an ideal; friction appeared instead in uneven enactment. Educators reported non-disclosure, disagreement with diagnosis, limited availability, and unsuccessful contact, while mothers described delayed, technical, or one-directional communication. The same behavior could receive competing adult explanations: silence as indifference, limited access, fear of labeling, disagreement, or distrust. Because educators and mothers were not paired and interactions were not observed, the analysis supports relational ambiguity rather than a complete model of why partnership fails.

### 4.4. What the Data Do—And Do Not—Show About Context

The contextual argument requires a strict distinction between interview material and contextual scholarship. Participants directly described a limited set of context-relevant issues: concerns about diagnosis and labeling, mistrust or non-disclosure, inaccessible professional or written communication, and exclusion from decisions. These accounts can be interpreted in relation to scholarship on stigma, communicative accessibility, trust, and institutional authority (Daley & Rappolt-Schlichtmann, 2018; Haft et al., 2023; Lucia & Soffer, 2020).

By contrast, Arabic-medium schooling, central Ministry supervision, curriculum structure, predominantly Arab staffing, national minority positioning, community proximity, shared language, and privacy or social visibility are features of the setting described in prior scholarship and official sources (Arar et al., 2014; Central Bureau of Statistics, 2018; Maagan & Blass, 2026; OECD, 2026). The interviews did not independently demonstrate how each feature shaped partnership. The manuscript therefore does not present them as participant findings.

The basic themes are not claimed to be unique to Arab education in Israel. The contribution is context-bounded rather than context-exclusive: these educator constructions were produced within Arab education, and some participant material concerned stigma, access, trust, and decision inclusion. Comparative research would be required to establish whether or how these processes differ across sectors or cultural settings.

### 4.5. Scope of the Contribution

The study offers a bounded, adult-centered account of how 13 educators narrated family–school partnership as attribution, communication, and professional action. Its central contribution is the distinction among contact, communication, co-interpretation, and decision inclusion, together with an account of the institutional conditions under which family knowledge may or may not influence professional understanding and action.

## 5. Participant-Informed Considerations for Practice and Inquiry

This study does not provide an implementation framework, evidence-based guideline, or policy recommendation. The considerations below remain close to questions raised by participants’ accounts and require local deliberation, co-design, and empirical evaluation before adoption.

First, educator accounts invite reflection on what school contact is intended to accomplish. Participants distinguished crisis notification from contact that invites family knowledge before an interpretation or decision hardens. A school or team could use this distinction to examine its own communication, but the data do not identify an optimal frequency, channel, or protocol, and they do not show how parents experience unsolicited proactive contact.

Second, the interviews in which educators described family knowledge, a changed or confirmed understanding, and a specified response in that order (M01, M02, M03, M06, M07, M10; Section 3.2) illustrate a question for support planning: under what conditions can family knowledge genuinely revise an educator’s initial formulation or communication response? The interviews suggest the value of asking this question.

Third, the four mothers’ accounts raise a limited question about influence: are parents being consulted while interpretations and decisions remain open, or informed after closure? Their small and specific sample is insufficient for a general recommendation. It may nevertheless justify locally examining whether communication provides voice as well as information.

Finally, professional learning may use these accounts to reflect on adult attribution, diagnostic language, communicative accessibility, and educator defensiveness. Such reflection should not assume that parents uniformly want more contact or that one culturally responsive script fits all families. Future intervention, observational, and co-designed research is needed before these participant-informed considerations can be translated into practice or policy claims.

## 6. Limitations and Future Research

The primary dataset comprised 13 educators heterogeneous in professional role and school level. This variation supported examination of shared meanings across direct experience with inclusion support but not subgroup comparison. Although the researcher had no prior relationship with participants or intermediaries and no authority over them, recruitment through social media groups and snowball referrals may have favored people willing to discuss partnership or connected to similar circles. Invitations explicitly stated that participation or refusal would have no professional consequences, and challenge-focused questions sought less favorable examples; neither safeguard can eliminate self-selection or socially desirable presentation. Sample adequacy rests on the information-power appraisal in Section 2.2, not on saturation or representativeness (Braun & Clarke, 2021; Malterud et al., 2016).

The four mother interviews were small, unmatched, and demographically narrow: all concerned daughters, three in technological-school settings and one in an undocumented school context. Their accounts cannot represent parents, validate educator findings, or compare adults discussing the same student. They are used only to complicate school-centered interpretations of communication and influence. Future studies require diverse parents, fathers, younger age groups, different school tracks, and matched educator–parent–student cases.

The interview guide itself shaped the dataset. Asking how partnership “contributes” to belonging, emotional support, or participation foregrounded a positive relationship and likely elicited benefit-oriented narratives. Reflexive analysis can acknowledge and reinterpret that influence, but cannot remove it retrospectively. Future interviews should include open questions about when partnership is unnecessary, unwanted, pressuring, contested, or unsuccessful.

Students were neither interviewed nor observed, and family–school interactions were not observed. Educator descriptions of confidence, security, belonging, or participation are retrospective attributions. The cross-case display likewise records the order of elements in educator narratives, not an independently verified temporal sequence; its counts describe this dataset and do not estimate prevalence. Even reported behavioral changes cannot establish causality because no temporal, observational, or comparative design tested the proposed pathway. Future research should include student voice, direct observation, longitudinal designs, and explicit examination of alternative explanations.

The contextual claims are also limited. The interviews provide evidence concerning labeling, communication access, mistrust, and decision inclusion, but do not establish the effects of minority positioning, shared language, community proximity, privacy, governance, curriculum, or staffing. Comparative studies are needed to distinguish context-specific from more general partnership processes.

Analysis was conducted by one researcher whose professional and scholarly familiarity with the setting shaped the questions and interpretation. Reflexive transparency makes that role visible but does not eliminate it. Translation from Arabic to English may also affect nuance and culturally specific meaning; translation fidelity was supported by the researcher’s own comparison of each translated quotation against the original recording or transcript, rather than by independent bilingual validation, which would offer a stronger safeguard against translation drift (Squires, 2009). Future work could analyze key concepts in Arabic and use multilingual, multi-perspective teams to explore alternative interpretations.

## 7. Conclusions

This study offers an adult-centered interpretive account of how 13 educators in Arab education in Israel constructed family–school partnership through attribution, communication, and professional action. Educators described changing the timing and stance of contact and, in a defined subset of accounts, narrated family information, changed or confirmed understanding, and a response in that order. The cross-case counts make the distribution and limits of this pattern visible within the dataset.

The four unmatched mother interviews were collected to explore school communication, transparency, and involvement in decisions and were analyzed through a separate bounded contextual reading. Their descriptions of after-the-fact information, technical communication, and limited influence show why contact cannot be equated with partnership, but they neither generated themes nor triangulated the educator accounts.

The study’s contribution is the distinction among contact, communication, co-interpretation, and decision inclusion, and the institutional question of whether family knowledge can enter while interpretations and decisions remain open. Claims remain bounded to adult accounts and to the interview-supported issues of labeling, communicative accessibility, mistrust, and decision inclusion; wider features of Arab minority education provide context through the literature rather than functioning as findings.

## Figures and Tables

**Table 1:** Participant Profile.

Code	Group/Gender	Role	School Context	Years of Experience
M01	Educator/Female	Inclusion teacher	High school	5
M02	Educator/Female	Inclusion teacher	Elementary school	6
M03	Educator/Male	English teacher	Elementary school	20
M04	Educator/Male	Homeroom teacher	Elementary school	5
M05	Educator/Male	Special education teacher	Elementary school; integrated special education	10
M06	Educator/Female	Inclusion teacher	Middle school	4
M07	Educator/Female	Inclusion teacher	Middle school	7
M08	Educator/Female	Inclusion teacher	Middle school	13
M09	Educator/Female	Special education teacher	Elementary school	15
M10	Educator/Female	Homeroom teacher	Elementary school	14
M11	Educator/Female	Special education teacher	Elementary school	11
M12	Educator/Female	Special education teacher	Middle school; special remedial class	14
M13	Educator/Female	Homeroom teacher	Elementary school	5
P01	Mother/Female	Mother of a female student	Technological school	Not applicable
P02	Mother/Female	Mother of a female student	Technological school	Not applicable
P03	Mother/Female	Mother of a female student	Technological school	Not applicable
P04	Mother/Female	Mother of a female student	Not documented	Not applicable

Note. M = teacher/educator interview; P = parent/mother interview. Gender is reported as documented in the interview records and transcripts. All participants were recruited from Arab education contexts in Israel. The table reports only non-identifying information relevant to the study. More specific locality, religious/community affiliation, age, and school-identifying details are withheld to protect confidentiality in a small and contextually identifiable sample. The mother interviews were unmatched and were analyzed separately from the educator themes.

**Table 2:** Educator-Derived Themes, Interpretive Processes, and Empirical Boundaries.

Theme	Central Analytic Meaning	Main Empirical Emphasis
From Crisis Contact to Relational Trust: Changing the Terms of Engagement	Educators distinguished contact that summons parents after difficulty from contact that changes timing, tone, purpose, and communicative access before crisis.	Adult-level process: Timing, channel, stance, and positioning of parents; the theme concerns how educators organized contact.
Parents as Co-Interpreters: Family Knowledge, Reattribution, and Response	Educators positioned family knowledge as capable of revising school-centered explanations of difficulty. Reported adaptation of response was a narrower subpattern.	Cross-case display: Both elements described in five accounts (six under the broader confirmatory criterion); one response-only, one reattribution-only, and five with no qualifying episode. Counts describe dataset distribution, not prevalence or theme importance.
Coordination as an Educator Attribution: Adult Alignment, Visibility, and Participation	Educators retrospectively linked coordinated adult action to changes they noticed in participation and to psychological meanings they inferred for students.	Educator attribution, not an observed causal mechanism. Participation was sometimes described behaviorally; belonging and security remained adult inferences.

Note. The table reports themes generated from the 13 educator interviews. The four unmatched mother interviews were examined separately and are reported after the educator themes.

**Table 3:** Cross-Case Descriptive Display of Family Information, Reattribution, and Response Accounts (Theme 2).

Participant	Family Information in Account	Changed/Confirmed Understanding	Changed Response	Later Change	Account Pattern
M01	Home also showed the symptoms; parents’ own framing was “just shyness”	Described (confirmatory)	Described—safe space, participation choice, written alternative	Yes—gradual risk-taking, small-group speech	Both (broader)
M02	Student’s strength in technology/programming	Described (strength reframing)	Described—technology role in school project	Yes—presented work in class	Both (strict)
M03	Mother’s non-disclosed illiteracy	Described	Described—written to telephone contact	Yes—greater maternal involvement	Both (strict)
M04	General knowledge of history/personality/environment	No specific episode	Not described	—	Neither
M05	What worked at home	Not described	Described—changed learning plan	Not specified	Response only
M06	Home pressures affecting concentration	Described	Described—coordinated plan and small goals	Implied (ongoing plan)	Both (strict)
M07	Complex emotional situation at home	Described	Described—unified response and calming cue	Implied (ongoing plan)	Both (strict)
M08	General partnership commentary	No specific episode	Not described	—	Neither
M09	General description of changed communication practice	No specific episode	Not described	—	Neither
M10	Caregiving role, cooking, writing poetry	Described (deficit framing broadened)	Described—new relationship and classroom responsibility	Implied	Both (strict)
M11	Sensory sensitivities, routines, music	Described	Not described	—	Understanding only
M12	(Disclosure came from the student, not a parent)	Not applicable—family-source criterion not met	—	—	Neither
M13	General communication constraints	No specific episode	Not described	—	Neither

Note. The display summarizes the order in which educators retrospectively narrated family information, changed or confirmed understanding, and response; it does not independently establish a temporal or causal pathway. “Both (strict)” requires a revised understanding plus a concrete response (M02, M03, M06, M07, M10). The broader criterion also includes confirmatory understanding (M01). Partial rows contain only one element. M12 has no qualifying episode because the disclosure originated with the student rather than a family member. Counts describe this dataset’s distribution, not prevalence, theme importance, or inter-rater reliability.

## Data Availability

The data are not publicly available due to confidentiality and privacy considerations related to the interview transcripts. Further inquiries may be directed to the author.
